# Biofilm Formation Ability of ESBL/pAmpC-Producing *Escherichia coli* Isolated from the Broiler Production Pyramid

**DOI:** 10.3390/antibiotics12010155

**Published:** 2023-01-11

**Authors:** Andrea Laconi, Roberta Tolosi, Ilias Apostolakos, Alessandra Piccirillo

**Affiliations:** 1 Department of Comparative Biomedicine and Food Science, University of Padua, 35020 Legnaro, PD, Italy; 2 Dairy Research Institute, Hellenic Agricultural Organization “DIMITRA”, 45221 Ioannina, Greece

**Keywords:** ESBL/pAmpC *E. coli*, biofilm, biofilm-associated genes, antimicrobial resistance

## Abstract

*Escherichia coli* able to produce extended spectrum β-lactamases (ESBLs) and plasmid-mediated AmpC β-lactamases (pAmpCs) represents a serious threat to public health, since these genes confer resistance to critically important antimicrobials (i.e., third generation cephalosporins) and can be transferred to non-resistant bacteria via plasmids. *E. coli* are known to be able to form a biofilm, which represents a favorable environment for the exchange of resistance determinants. Here, we assessed the ability of 102 ESBL/pAmpC-producing *E. coli* isolated from the broiler production pyramid to form a biofilm and to identify genetic factors involved in biofilm formation. All but one of the ESBL/pAmpC-producing *E. coli* were able to form a biofilm, and this represents a great concern to public health. *E. coli* belonging to phylogroups D, E, and F, as well as strains harboring the *bla_CTX-M-type_* gene, seem to be associated with an increased biofilm capability (*p* < 0.05). Furthermore, virulence genes involved in adherence and invasion (i.e., *csgBAC*, *csgDEFG*, *matABCDEF*, and *sfaX*) seem to enhance biofilm formation in *E. coli*. Efforts should be made to reduce the presence of ESBL/pAmpC- and biofilm-producing *E. coli* in the broiler production pyramid and, therefore, the risk of dissemination of resistant bacteria and genes.

## 1. Introduction

In recent years, *Escherichia coli* antibiotic resistance has increased significantly, posing a threat to animal and human health [1]. Among the resistance mechanisms emerging in this species, extended spectrum β-lactamases (ESBLs) and plasmid-mediated AmpC β-lactamases (pAmpCs) represent a great concern for public health. Indeed, they hinder resistance to third generation cephalosporins, which are considered by the World Health Organization (WHO) as Highest Priority Critically Important Antimicrobials (HPCIA), essential for the treatment of serious bacterial infections and one of the few alternatives to the treatment of sepsis and respiratory tract infections in various animal species, including humans. Furthermore, ESBL/pAmpC genes are located on mobile genetic elements (MGEs), allowing their exchange between bacteria [2].

*E. coli* persisting in the environment and especially those residing within biofilms can also contribute to the spread of antimicrobial resistance. Indeed, biofilm is known to be a hot spot for horizontal gene transfer (HGT) within and between bacterial species [3,4]. Biofilms are heterogeneous structures formed by bacterial populations enclosed in an extracellular matrix and able to colonize abiotic and biotic surfaces [5]. Bacteria living within biofilms show several advantages compared to those in planktonic form, such as greater protection against chemical, biological, and mechanical agents, thus surviving common disinfection and cleaning treatments [6].

The presence of ESBL/pAmpC-producing *E. coli* (ESBL/pAmpC-EC) has been detected at different levels of the poultry production chain, and the consumption of contaminated chicken meat has been suggested as one of the main sources of human infection caused by resistant *E. coli* [7]. Similarly, biofilm-producing *E. coli* have been identified on the surface of poultry farms, poultry meat processing facilities, and poultry meat packaging, showing themselves to be highly resistant to disinfection and sanitation procedures, and, therefore, representing a threat to consumers’ health [8].

In the present study, the biofilm formation ability of 102 ESBL/pAmpC-producing *E. coli* strains isolated from different stages of the broiler production pyramid was evaluated in vitro by using a microtiter assay and correlated with environmental and genetic factors that could be involved in biofilm formation.

## 2. Results

### 2.1. Biofilm Production Assay

**Figure 1:**
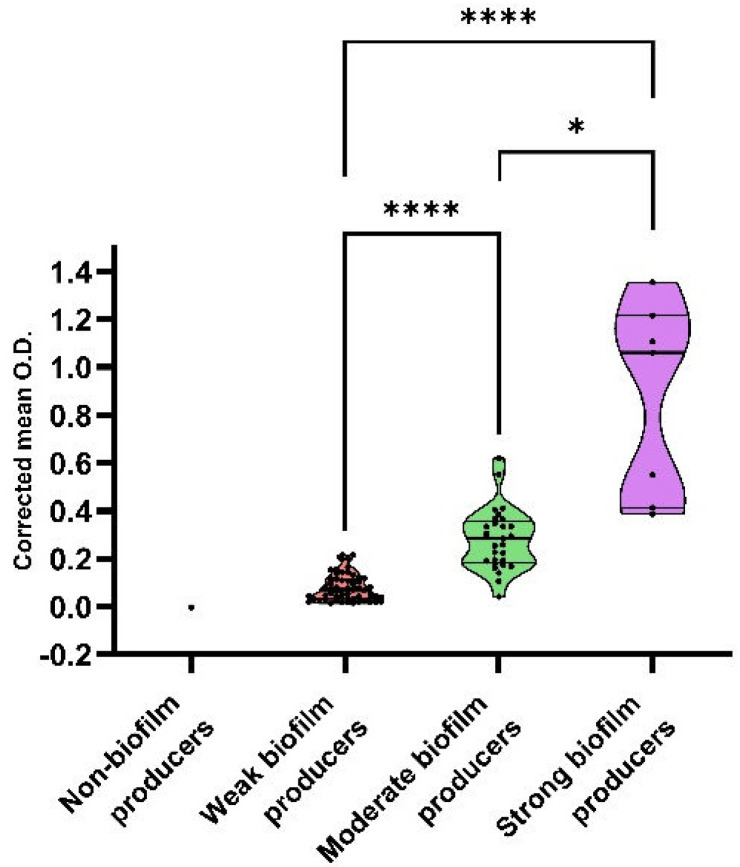
Corrected mean optical density (O.D.) values of the different biofilm classes. Lines within violin plots represent median, 25th, and 75th percentile. *p* < 0.05 is shown as * and *p* < 0.0001 as ****.

All but one of the ESBL/pAmpC-EC investigated was able to form biofilm. Based on the results of the mean optical densities (O.D.), strains were classified as strong (*n* = 7, 6.86%, 95% confidence of interval (CI) 1.87–11.85%), moderate (*n* = 31, 30.39%, 95% CI 21.31–39.47%), weak (*n* = 63, 61.76%, 95% CI 52.17–71.36%), and non-biofilm producers (*n* = 1, 0.98%, 95% CI 0.00–2.92%). The mean O.D. values for each group are depicted in Figure 1, and statistically significant differences in O.D. between groups were observed (*p* < 0.05).

### 2.2. Distribution of Biofilm-Producing ESBL/pAmpC E. coli along the Poultry Production Pyramid

The frequency of biofilm classes among sampling sites (i.e., production chains and stages of the production pyramid) is listed in Table 1. 

**Figure 2:**
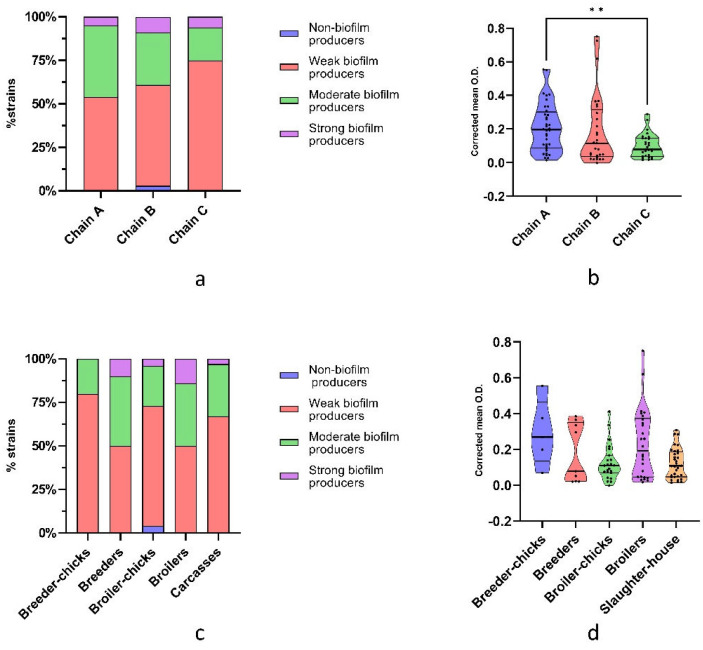
(**a**) Distribution of biofilm formation classes, presented as cumulative percentage of strains, and (**b**) corrected mean O.D. among production chains. (**c**) Distribution of biofilm formation classes, presented as cumulative percentage of strains, and (**d**) corrected mean O.D. among production stages. Lines within violin plots represent median, 25th, and 75th percentile. *p* < 0.01 is shown as **.

Biofilm-producing ESBL/pAmpC-EC were identified in all production chains, and even though they were not statistically significant, some differences in the distribution of the biofilm classes between chains was observed (Figure 2a). Indeed, chain C showed the highest prevalence of weak producers (75% vs. 54.05% and 57.58% of chains A and B, respectively), chain A of moderate producers (40.54% vs. 30.30% and 18.75% of chains B and C, respectively), and chain B of strong producers (9.09% vs. 5.41% and 6.25% of chains A and C, respectively). Multiple comparisons between the corrected mean O.D. revealed a significant difference (*p* = 0.0085) between chain A and chain C (Figure 2b), confirming the association of the latter with weak biofilm-producing strains.

No strong producers were identified in breeder chicks, and the prevalence of moderate and strong biofilm producers seemed to increase in adult birds (Figure 2c); however, no significant differences between production stages were identified. Similarly, no significant differences in corrected mean O.D. were detected between production stages (Figure 2d). 

### 2.3. Genetic Characteristic of Biofilm-Producing ESBL/pAmpC E. coli

The ability to produce the biofilm of the investigated ESBL/pAmpC-EC seems to be associated with the genotype. In detail, weak biofilm production was observed more frequently in phylogroup A compared to phylogroup B1 (*p* = 0.023) and in phylogroup B2 than in phylogroups B1 (*p* = 0.0068), E (*p* = 0.0464), and F (*p* = 0.0361). On the other hand, moderate biofilm-producing strains were more frequent in phylogroup B1 than in phylogroups A and B2 (*p* = 0.0458 and *p* = 0.0235, respectively). Notably, no strong biofilm producers were detected in phylogroups A, B2, and C, and no associations were identified between phylogroups and strong biofilm-producing strains. The frequency of biofilm classes among phylogroups is reported in Table 2.

**Figure 3:**
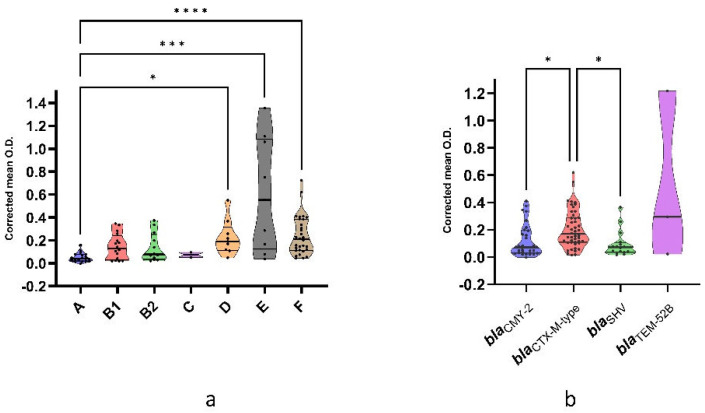
(**a**) Corrected mean O.D. among phylogroups. (**b**) Corrected mean O.D. among strains harboring different ESBL/pAmpC genes. Lines within violin plots represent median, 25th, and 75th percentile. *p* < 0.05 is shown as *, *p* < 0.005 as ***, and *p* < 0.0001 as ****.

The statistical analysis of the corrected mean O.D. seems to confirm the association between strains belonging to phylogroup A and the limited ability to form biofilms, since differences were observed between this phylogroup and phylogroups D, E, and F (*p* = 0.0104, *p* = 0.0001, and *p* < 0.0001, respectively) (Figure 3a).

Interestingly, correlations were identified between biofilm producing classes and the frequency of ESBL/pAmpC-encoding genes. Indeed, strains harboring *bla_CMY-2_* were associated with weak biofilm production (*p* = 0.0326), while *bla_CTX-M-type_* gene was associated with moderate biofilm production ability (*p* = 0.0436). The distribution of biofilm classes among strains harboring different ESBL/pAmpC genes is reported in Table 2. According to the association between ESBL/pAmpC genes and biofilm classes, the corrected mean O.D. was significantly higher in strains carrying the *bla_CTX-M-type_* gene than those harboring *bla_CMY-2_* and *bla_SHV_* genes (*p* = 0.0253 and *p* = 0.0144, respectively) (Figure 3b). 

### 2.4. Analysis of Virulence Genes Associated with Biofilm Formation

**Figure 4:**
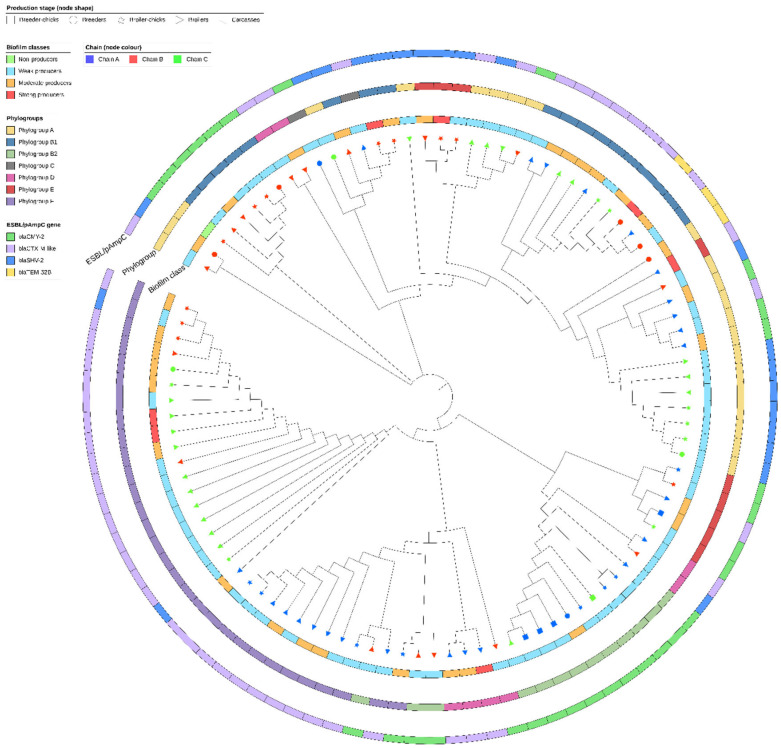
Hierarchical clustering analyses based on average linkage distance and Euclidean distance measurement methods showing the clustering pattern of ESBL/pAmpC-EC strains (*n* = 102) and variable virulence genes (*n* = 21). Nodes shape and color indicate the broiler production stage and production chain, respectively. The inner stripe indicates the biofilm classes. The middle stripe indicates the phylogroups. The outer ring specifies the ESBL/pAmpC genes identified.

Out of 70 virulence genes associated with biofilm formation, adherence, and cell invasion (Table S1), 29 were detected among the 102 strains included in this study. Nine genes (i.e., *csgB*, *csgC*, *csgD*, *csgE*, *csgF*, *fimF*, *fimG*, *hha*, and *papC)* were identified in all strains, while the remaining genes were detected only in some strains (min = 1 and max = 100). No associations were identified between the number of genes and the ability to form biofilm, and strains belonging to different biofilm classes harbored a similar number of virulence genes (mean = 20.63 and 95% CI 19.84–21.43, mean = 20.52 and 95% CI 19.31–21.72, and mean = 20.86 and 95% CI 18.56–23.15, for weak, moderate, and strong producers, respectively). Hierarchical clustering analyses based on the frequency of 21 variable virulence genes did not show any clear cluster of strains according to the explanatory variables (i.e., stage, chain, phylogroup, ESBL/pAmpC gene, and biofilm class) (Figure 4).

**Figure 5:**
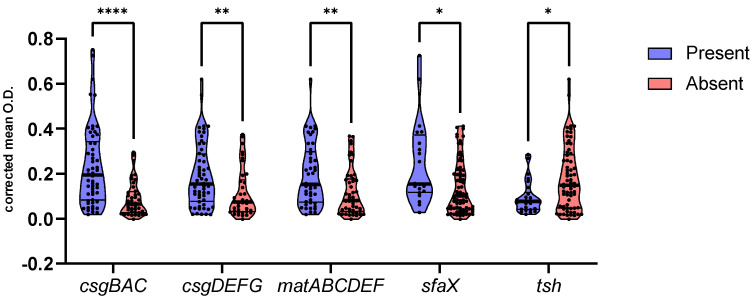
Corrected mean O.D. of strains harboring (blue) and lacking (red) operons csgBAC, csgDEFG, and matABCDEF and genes sfaX and tsh. Lines within violin plots represent median, 25th, and 75th percentile. *p* < 0.05 is shown as *, *p* < 0.01 as **, and *p* < 0.0001 as ****.

Statistical analysis showed a significant reduction in corrected mean O.D. in strains harboring *tsh* gene (*p* = 0.015), while the presence of operons *csgBAC* (*p* < 0.0001), *csgDEFG* (*p* = 0.0082), *matABCDEF* (*p* = 0.0082), and gene *sfaX* (*p* = 0.0127) was associated with increased corrected mean O.D. (Figure 5). No other statistically significant differences were identified between the frequency of virulence genes and the corrected mean O.D. Any multivariable analysis was precluded by the high collinearity between the investigated genetic components.

## 3. Discussion

Biofilm production provides bacteria with remarkable survival advantages, allowing protection against mechanical, chemical, and biological agents, and it encourages the transfer of antimicrobial resistance genes between bacteria. Indeed, bacteria that reside in a biofilm within the water pipeline or on the surfaces of livestock productions are less susceptible to detergent and antimicrobial treatments.

The vast majority (98.03%) of ESBL/pAmpC-EC strains isolated from the three different chains of an integrated Italian poultry company were able to produce biofilm, confirming the extent of this issue in *E. coli* carrying ESBL/pAmpC genes [9,10].

Of concern, biofilm-producing ESBL/pAmpC-EC seem to be disseminated throughout the broiler production pyramid; although a higher prevalence of moderate and strong producing strains was observed in adult birds compared to chicks. These findings, together with differences in the ability to form the biofilms of strains isolated from different production chains, seem to suggest that biofilm-producing ESBL/pAmpC-EC emerged in the poultry pyramid as result of the combinatory effect of vertical transmission (i.e., from generation to generation), environmental contamination, within and between farms transmission, and resistant bacteria proliferation, as previously hypothesized [11]. Furthermore, all ESBL/pAmpC-EC isolated from carcasses were able to form biofilms; considering the direct link between meat contaminated with ESBL/pAmpC-EC and human exposure; this represents a concern for human health [7,12]. Indeed, surface contamination with biofilm-producing ESBL/pAmpC-EC during the manipulation of the carcasses might promote the establishment of persistent biofilms in slaughterhouses, contributing to the dissemination of bacteria resistant to third generation cephalosporins through the food chain, as well as to operators.

Relations between biofilm classes and genetic factors were also identified. Indeed, strains belonging to phylogroups A and B2 seemed to have lower ability to form biofilms compared to other phylogroups (B1, D, E, and F). High biofilm production was detected for strains belonging to phylogroups associated with extra-intestinal pathogenicity (D, E, and F), while commensal phylogroup A showed the lowest correct mean O.D.; however, this pattern was not observed for phylogroups B2 (associated with weak producers) and B1 (associated with moderate producers), considered pathogenic and commensal, respectively. Although an association between phylogroups and biofilm formation ability might be plausible, no direct links between biofilm production and extra intestinal pathogenicity can be established. Strains harboring *bla_CTX-M-type_* gene seem to be associated with higher biofilm production in comparison to those carrying *bla_CMY-2_* and *bla_SHV_* genes. This finding seems to agree with previous observations, indicating *E. coli* harboring *bla_CTX-M-type_* gene as rapid and effective biofilm developers [13].

In this study, we also investigated the associations between biofilm production and several virulence factors encoding adhesins, invasins, and quorum sensing that might be involved in the initial bacterial adhesion process to surfaces and in the establishment of the mature biofilm. The presence of operons *csgBAC* and *csgDEFG* was correlated with increased biofilm production. Indeed, *csgBAC* genes encode curli fibers, which have been linked to biofilm formation in *E. coli* and other enterobacteria [14], while proteins encoded by *csgDEFG* genes regulate the transcription from the *csgBAC* promoter (CsgD) and are involved in curli assembly and stability (CsgE, CsgF, and CsgG) [15]. Previous studies suggest that curli fibers may play a key role during infection, particularly in the attachment and invasion of host cells [16], interaction with host proteins [17], and activation of the immune system [18], highlighting the risk for human health posed by biofilm-producing ESBL/pAmpC-EC. Operon *matABCDEF* is known to be involved in the early stages of biofilm formation, and defective *E. coli* has been shown to have a reduced ability to form biofilms [19]. Accordingly, in our study, strains carrying the entire operon showed higher biofilm production compared to those lacking one or more of these genes. Increased biofilm formation was also observed in the presence of the *sfaX* gene. Although the function of the encoded protein has not yet been elucidated, it seems to have a regulatory role affecting the expression of surface structures, such as type 1 fimbriae and flagella [20], which are associated with an increased biofilm formation capability in *E. coli* [21]. Our data seem to suggest that the SfaX protein might enhance biofilm production, possibly by promoting fimbrial expression [22], although transcription or expression studies are needed to support such a hypothesis. *Tsh* gene, which encodes a temperature-sensitive hemagglutinin member of the autotransporter group of proteins, was the only gene associated with a reduction in biofilm formation capability. *Tsh* has been identified in *E. coli* isolated from different sources [23] and has been associated with extraintestinal infection and virulence of Avian Pathogenic *E. coli* (APEC) [24]. Despite its role in APEC virulence and the fact that autotransporter proteins are recognized to be implicated in different virulence functions, including adherence to and invasion of host cells, our findings seem in agreement with previous observations, suggesting that the Tsh protein does not enhance biofilm formation [25,26]. All strains lacked the *mrkABCDF* operon, encoding for the type 3 fimbriae, which enhances biofilm formation [27], but possessed the *pgaC* gene, encoding for the synthesis of the PGA polymer, which is involved in the early stages of biofilm formation by promoting the adhesion to abiotic surfaces [13]. Remarkably, all strains showed the *hha* gene encoding a protein involved in the suppression of fimbrial genes (*fim*) transcription and playing a dual role in the biofilm cycle. Indeed, its expression in planktonic *E. coli* can hinder initial biofilm formation, whereas within mature biofilms it can promote cell detachment from the biofilm, cell dispersal, and colonization of new ecological niches [28]. The study is limited to assessing the presence of biofilm formation-associated genes, but no transcription and/or expression analysis was carried out. Future studies should aim at investigating the expression of the identified genes, allowing us to improve the knowledge regarding the genetic factors influencing the biofilm formation ability of ESBL/pAmpC-EC.

## 4. Materials and Methods

### 4.1. Bacterial Strains

One hundred and two ESBL/pAmpC-producing *E. coli* isolated from three production chains (i.e., A, B and C) of an integrated broiler production company located in Northern Italy were included in this study (Table S2) [11]. Briefly, in each chain, cloacal swabs were first collected from broiler breeders at two time-points, i.e., at 1 day of age (breeder chicks) and 21 weeks of age (breeders), and then from the offspring in four fattening farms at 1 (broiler chicks) and 30 days of age (broilers). Carcasses were sampled at the slaughterhouse by rinsing the whole carcass with buffer peptone water. *E. coli* were screened for ESBL/pAmpC using a selective medium (Eosin Methylene Blue agar (Microbiol, Italy) supplemented with 1 mg/L cefotaxime (CTX-EMB) and incubated at 37 ± 0.5 °C for 20 ± 2 h; ESBL/pAmpC production was assessed by double-disk synergy according to CLSI guidelines [29]. Phylogroups characterization and antimicrobial resistance genes (ARGs) detection were carried out by multiplex PCR [11]. Strains were selected based on the production chain, production stage, ESBL/pAmpC gene, and phylogroup combinations, and underwent whole genome sequencing [30,31]. Raw reads were assembled using the Enterobase database (https://enterobase.readthedocs.io, accessed on 28 December 2020) and the obtained contigs were used for the comparative genomics analysis (Table S2).

### 4.2. Microtitre Plate Assay for the Detection and Quantification of Biofilm Formation

The biofilm formation was assessed using a microtiter plate assay based on the protocol published by Stefanovic et al. (2000). Each strain was tested in triplicate on two different experiments (six replicates per sample). In detail, *E. coli* strains were incubated in brain heart infusion (BHI) broth (Microbiol, Uta, Italy) at 37 ± 1 °C for 24 h. Subsequently, aliquots were diluted until reaching the turbidity of 1 on the McFarland scale, and 200 µL of each bacterial suspension were inoculated in triplicate into a 96-well sterile polystyrene microplate (Sarstedt, Numbrecht, Germany). In each plate, three wells were inoculated with sterile BHI as negative control. Plates were incubated on a shaker at 37 ± 1 °C for 24 h. After incubation, the bacterial suspensions were removed and wells were washed three times with 0.9% sodium chloride solution (250 µL). Bacteria were then fixed using methanol (200 µL) and incubated at room temperature for 15 min (min). Subsequently, bacteria were stained with 200 µL crystal violet (Fisher Scientific, Waltham, USA) for 5 min, then the dye was washed with running water and dried at room temperature. Eventually, the dye bound to the cells adhering to the wells was re-solubilized with 160 µL of 33% glacial acetic acid and plates were read three times at 5 min intervals using a spectrophotometer (Multiskan^TM^ GO, Fisher Scientific) at 570 nm. The O.D. value of each strain (O.D._S_) was calculated as the arithmetic mean of the absorbance of the replicates over the two experiments. Standard deviation and coefficient of variation (CV) were also calculated to assess the robustness of the data (CV < 0.5). O.D._S_ values were compared with the mean O.D. of the negative control (O.D._NC_), and strains were subsequently classified into four different categories: (1) non biofilm producers (O.D._S_ ≤ O.D._NC_); (2) weak biofilm producers (O.D._NC_ < O.D._S_ ≤ 2 × O.D._NC_); (3) moderate biofilm producers (2 × O.D._NC_ < O.D._S_ ≤ 4 × O.D._NC_); and (4) strong biofilm producers (O.D._S_ > 4 × O.D._NC_) [32].

### 4.3. Statistical Analysis and Comparative Genomics

Association between the explanatory variables (i.e., stage, chain, phylogroup, and ESBL/pAmpC gene) was assessed using chi-square and Fisher’s exact test.

Assembled contigs were screened against a dataset containing the sequences of genes associated with biofilm formation (Table S1) using MyDbFinder 2.0 (https://cge.food.dtu.dk/services/MyDbFinder/, accessed on 24 August 2022).

The correlation between the ability to form biofilms and the frequency of virulence-associated genes was assessed using hierarchical clustering based on the presence/absence of each gene using the pHeatmap package version 1.0.12 in R version 4.1.2 (https://www.r-project.org/, accessed on 3 November 2022). The corrected mean O.D. was calculated for each strain as the mean of six independent replicates, minus the O.D. of the negative control. Differences in the corrected mean O.D. between groups (i.e., chain, stage, phylogroup, and ESBL/pAmpC genes) were assessed using the non-parametric Kruskal–Wallis test, while the association between corrected mean O.D. and the frequency of biofilm-associated genes was investigated with the Mann–Whitney test. The statistical analyses were performed using GraphPad Prims v9.4.1 software (http://www.graphpad.com, accessed on 6 October 2022).

## 5. Conclusions

In the present study, the biofilm formation ability of *E. coli*-carrying ESBL/pAmpC genes isolated from the poultry sector was investigated. Remarkably, biofilm-producing ESBL/pAmpC-EC were isolated through the broiler production pyramid, and this represents a great risk for public health, since they might persist at various points of the food chain and reach humans via the manipulation and consumption of contaminated food. ESBL/pAmpC-EC in biofilms can also act as spreaders of genes conferring resistance to third generation cephalosporins, which are critically important antimicrobials. To reduce the risk of human exposure to biofilm-producing ESBL/pAmpC-EC, intervention and mitigation strategies should be employed in poultry farms (e.g., increased biosecurity and disinfection measures) and slaughterhouses (e.g., disinfection of the processing line) to avoid the colonization of birds from environmental sources and the cross-contamination of poultry meat, respectively. Strains harboring adhesion and invasion genes were associated with increased biofilm formation ability, but future studies should be carried out to establish their role in biofilm formation.

## Figures and Tables

**Table 1:** Ability to form biofilms by ESBL/pAmpC-EC according to production chain and production stage.

	Non-Biofilm Producers		Weak Producers		Moderate Producers		Strong Producers	
	Percentage (%)	95% (CI)	Percentage (%)	95% (CI)	Percentage (%)	95% (CI)	Percentage (%)	95% (CI)
Chain								
Chain A (*n* = 37)	0.00%	0.00%	54.05%	37.21–70.90%	40.54%	23.94–57.14%	5.41%	00.00–13.05%
Chain B (*n* = 33)	3.03%	0.00–9.20%	57.58%	39.78–75.37%	30.30%	13.75–46.85%	9.09%	0.00–19.44%
Chain C (*n* = 32)	0.00%	0.00%	75.00%	59.14–90.86%	18.75%	4.45–33.05%	6.25%	0.00–15.12%
Production stage								
Breeder chicks (*n* = 5)	0.00%	0.00%	80.00%	24.47–100%	20.00%	0.00–75.53%	0.00%	0.00%
Breeders (*n* = 10)	0.00%	0.00%	50.00%	12.30–87.70%	40.00%	3.06–76.94%	10.00%	0.00–32.62%
Broiler chicks (*n* = 26)	3.85%	0.00–11.77%	69.23%	50.22–88.24%	23.08%	5.72–40.43%	3.85%	0.00–11.77%
Broilers (*n* = 28)	0.00%	0.00%	50.00%	30.26–69.74%	35.71%	16.79–54.64%	14.29%	0.47–28.10%
Carcasses (*n* = 33)	0.00%	0.00%	66.67%	49.69–83.64%	30.30%	13.75–46.85%	3.03%	0.00–9.20%

**Table 2:** Ability to form biofilms by ESBL/pAmpC-EC, according to phylogroup and ESBL/pAmpC gene.

	Non-Biofilm Producer		Weak Producers		Moderate Producers		Strong Producers	
	Percentage (%)	95% (CI)	Percentage (%)	95% (CI)	Percentage (%)	95% (CI)	Percentage (%)	95% (CI)
Phylogroup								
Phylogroup A (*n* = 22)	4.55%	0.00–14.00%	77.27%	58.25–96.29%	18.18%	0.68–35.68%	0.00%	0.00%
Phylogroup B1 (*n* = 18)	0.00%	0.00%	38.89%	13.94–63.83%	50.00%	24.41–75.59%	11.11%	0.00–27.19%
Phylogroup B2 (*n* = 12)	0.00%	0.00%	91.67%	73.33–100.0%	8.33%	0.00–26.67%	0.00%	0.00%
Phylogroup C (*n* = 2)	0.00%	0.00%	100.00%	100.00%	0.00%	0.00%	0.00%	0.00%
Phylogroup D (*n* = 9)	0.00%	0.00%	55.56%	15.04–96.07%	33.33%	0.00–71.77%	11.11%	0.00–36.73%
Phylogroup E (*n* = 9)	0.00%	0.00%	44.44%	3.93–84.86%	33.33%	0.00–71.77%	22.22%	0.00–56.12%
Phylogroup F (*n* = 30)	0.00%	0.00%	56.67%	37.85–75.49%	36.67%	18.36–54.97%	6.67%	0.00–16.14%
ESBL/pAmpC								
*bla_CMY-2_* (*n* = 30)	3.33%	0.00–10.15%	76.67%	60.60–92.73%	20.00%	4.81–35.19%	0.00%	0.00%
*bla_CTX-M-type_* (*n* = 49)	0.00%	0.00%	51.02%	36.51–65.53%	40.82%	26.55–55.08%	8.16%	0.22–16.11%
*bla_SHV_* (*n* = 20)	0.00%	0.00%	70.00%	48.00–92.00%	20.00%	0.79–39.21%	10.00%	0.00–24.41%
*bla_TEM-52B_* (*n* = 2)	0.00%	0.00%	0.00%	0.00%	50.00%	0.00–100.00%	50.00%	0.00–100.00%

## Data Availability

Not applicable.

## Supplementary Materials

The following supporting information can be downloaded at: https://www.mdpi.com/article/10.3390/antibiotics12010155/s1, Table S1: List of biofilm-associated genes identified in the investigate ESBL/pAmpC-producing *E. coli*; Table S2: Dataset of biofilm-associated genes used in this study.
